# HbA_1c_ Alone Is a Poor Indicator of Cardiometabolic Risk in Middle-Aged Subjects with Pre-Diabetes but Is Suitable for Type 2 Diabetes Diagnosis: A Cross-Sectional Study

**DOI:** 10.1371/journal.pone.0134154

**Published:** 2015-08-12

**Authors:** Seán R. Millar, Ivan J. Perry, Catherine M. Phillips

**Affiliations:** HRB Centre for Health and Diet Research, Department of Epidemiology and Public Health, University College Cork, Cork, Ireland; University of Padova, ITALY

## Abstract

**Objectives:**

Glycated haemoglobin A_1c_ (HbA_1c_) measurement is recommended as an alternative to fasting plasma glucose (FPG) for the diagnosis of pre-diabetes and type 2 diabetes. However, evidence suggests discordance between HbA_1c_ and FPG. In this study we examine a range of metabolic risk features, pro-inflammatory cytokines, acute-phase response proteins, coagulation factors and white blood cell counts to determine which assay more accurately identifies individuals at increased cardiometabolic risk.

**Materials and Methods:**

This was a cross-sectional study involving a random sample of 2,047 men and women aged 46-73 years. Binary and multinomial logistic regression were employed to examine risk feature associations with pre-diabetes [either HbA_1c_ levels 5.7-6.4% (39-46 mmol/mol) or impaired FPG levels 5.6-6.9 mmol/l] and type 2 diabetes [either HbA_1c_ levels >6.5% (>48 mmol/mol) or FPG levels >7.0 mmol/l]. Receiver operating characteristic curve analysis was used to evaluate the ability of HbA_1c_ to discriminate pre-diabetes and diabetes defined by FPG.

**Results:**

Stronger associations with diabetes-related phenotypes were observed in pre-diabetic subjects diagnosed by FPG compared to those detected by HbA_1c_. Individuals with type 2 diabetes exhibited cardiometabolic profiles that were broadly similar according to diagnosis by either assay. Pre-diabetic participants classified by both assays displayed a more pro-inflammatory, pro-atherogenic, hypertensive and insulin resistant profile. Odds ratios of having three or more metabolic syndrome features were also noticeably increased (OR: 4.0, 95% CI: 2.8-5.8) when compared to subjects diagnosed by either HbA_1c_ (OR: 1.4, 95% CI: 1.2-1.8) or FPG (OR: 3.0, 95% CI: 1.7-5.1) separately.

**Conclusions:**

In middle-aged Caucasian-Europeans, HbA_1c_ alone is a poor indicator of cardiometabolic risk but is suitable for diagnosing diabetes. Combined use of HbA_1c_ and FPG may be of additional benefit for detecting individuals at highest odds of type 2 diabetes development.

## Introduction

The prevalence of type 2 diabetes, a chronic disease which causes significant mortality, has increased considerably in world populations, representing a major public health issue [1]. Diabetes is associated with a clustering of cardiometabolic features including obesity, dyslipidaemia, hypertension, insulin resistance, chronic low-grade inflammation [2, 3], and may lead to severe cardiovascular complications [4].

Pre-diabetes, a condition defined by glycaemic profiles that are higher than normal but which do not meet thresholds for diabetes, is a strong risk factor for type 2 diabetes and related complications [5]. The American Diabetes Association (ADA) classifies type 2 diabetes as a fasting plasma glucose (FPG) level ≥7.0 mmol/l and pre-diabetes as impaired FPG levels between 5.6–6.9 mmol/l [2]. In 2009 the International Expert Committee recommended glycated haemoglobin A_1c_ (HbA_1c_) as an alternative marker [6], and in 2010 the ADA introduced HbA_1c_ cut-points of ≥6.5% (≥48 mmol/mol) for diabetes diagnosis and between 5.7–6.4% (39–46 mmol/mol) as a criterion to identify individuals at a high-risk state of developing diabetes [2]. Perceived benefits of the use of HbA_1c_ measurement, over FPG, include greater pre-analytical stability, lower biological variability and that the assay may be performed in non-fasting blood samples [7, 8]. However, use of HbA_1c_ as a screening tool has been controversial, with research showing discordance between HbA_1c_ and FPG [9–12], and several studies suggesting that factors such as age or ethnicity may influence diagnostic performance [13–15].

The aim of this study was to compare the metabolic profiles in subjects with pre-diabetes and type 2 diabetes, using ADA-recommended HbA_1c_ and FPG diagnostic thresholds, in a random sample of 2,047 middle-aged men and women. In particular, we examined a range of diabetes risk factors, metabolic syndrome (MetS) features, pro-inflammatory cytokines, acute-phase response proteins, coagulation factors and white blood cell (WBC) counts to determine which assay more accurately identifies individuals at increased cardiometabolic risk.

## Materials and Methods

### Study population

The Cork and Kerry Diabetes and Heart Disease Study (Phase II) was a single centre, cross-sectional study conducted between 2010 and 2011. A random sample was recruited from a large primary care centre in Mitchelstown, County Cork, Ireland. The Livinghealth Clinic serves a population of approximately 20,000 Caucasian-European subjects, with a mix of urban and rural residents. Stratified sampling was employed to recruit equal numbers of men and women from all registered attending patients in the 46–73 year age group. In total, 3,807 potential participants were selected from the practice list. Following the exclusion of duplicates, deaths, and subjects incapable of consenting or attending appointment, 3,051 were invited to participate in the study and of these, 2,047 (49.2% male) completed the questionnaire and physical examination components of the baseline assessment (response rate: 67.1%). The status of non-responders included individuals refusing to participate (59.4%) and those who did not reply (40.6%). Male subjects accounted for 53.7% of non-responders while 43.5% (vs. 42.8% of responders) were >60 years of age. Details regarding the study design, sampling procedures and methods of data collection have been reported previously [16].

Ethics committee approval conforming to the Declaration of Helsinki was obtained from the Clinical Research Ethics Committee of University College Cork. A letter signed by the contact GP in the clinic was sent out to all selected participants with a reply slip indicating acceptance or refusal. All subjects gave signed informed consent, including permission to use their data for research purposes.

### Clinical and laboratory procedures

All study participants attended the clinic in the morning after an overnight fast and blood samples were taken on arrival. Data on age, gender, family diabetes history, physician-diagnosed type 2 diabetes and prescription (Rx) medication use were gathered through a self-completed General Health Questionnaire. Triglyceride and high density lipoprotein cholesterol (HDL-C) levels were measured by Cork University Hospital Biochemistry Laboratory on Olympus 5400 biochemistry analysers with Olympus reagents using standardised procedures and fresh samples (Olympus Diagnostica GmbH, Hamburg, Germany). Fasting glucose concentrations were determined using a glucose hexokinase assay (Olympus Life and Material Science Europa Ltd., Lismeehan, Co. Clare, Ireland) and HbA_1c_ levels were measured in the haematology laboratory on an automated high-pressure liquid chromatography instrument Tosoh G7 [Tosoh HLC-723 (G7), Tosoh Europe N.V, Tessenderlo, Belgium]. Serum insulin, c-reactive protein (CRP), tumour necrosis factor alpha (TNF-α), interleukin 6 (IL-6), adiponectin, leptin, resistin and plasminogen activator inhibitor-1 (PAI-1) were assessed using a biochip array system (Evidence Investigator; Randox Laboratories, UK). Complement component 3 (C3) was measured by immunoturbidimetric assay (RX Daytona; Randox Laboratories). White blood cell counts were determined by flow cytometry technology as part of a full blood count.

Three independent measurements of systolic and diastolic blood pressure (BP) were obtained with the subject in a seated position using an Omron M7 digital sphygmomanometer (Omron Healthcare Co. Ltd., Japan). The mean of the second and third readings was considered to be a subject’s BP. The weight and height of each participant were measured to the nearest 0.1 kg and 0.1 cm respectively. Portable electronic Tanita WB-100MA weighing scales (Tanita Corporation, IL, USA) were placed on a firm, flat surface and were calibrated weekly to ensure accuracy. Height was measured using a portable Seca Leicester height/length stadiometer (Seca, Birmingham, UK) and body mass index (BMI) was calculated as weight divided by the square of height. A BMI ≥30 kg/m^2^ was classified as obese. Waist circumference (WC) was measured between the lowest rib and iliac crest on bare skin. Subjects were instructed to breathe in, and then out, and to hold their breath while measurement was made to the nearest 0.1 cm using a Seca 200 measuring tape. Two independent measurements of WC were taken and the mean of the two was used in analysis. Central obesity was defined as a WC level ≥102 cm for males and ≥88 cm for females.

### Classification of biochemical and blood pressure measurements

Lipid, lipoprotein and BP measurements were categorised according to National Cholesterol Education Program Adult Treatment Panel III (NCEP: ATP III) guidelines [17]. Abnormal metabolic risks were defined as high triglycerides ≥1.7 mmol/l and low HDL-C (<1.03 mmol/l in males or <1.29 mmol/l in females). Dyslipidaemia was determined according to both high triglyceride and low HDL-C levels. Elevated BP was classified as systolic BP ≥130 mmHg and/or diastolic BP ≥85 mmHg or Rx anti-hypertensive medication use. High serum insulin was defined as a level equal to or above the 75^th^ percentile in the study sample. Metabolic syndrome was determined according to a modified version of the NCEP: ATP III criterion, substituting serum insulin 75^th^ percentile for impaired FPG. Three or more MetS features (≥3 MetS) was characterised as any combination of the following: obesity defined by WC, high triglyceride levels, low HDL-C, elevated BP and high insulin concentrations. According to ADA guidelines, pre-diabetes was classified as elevated HbA_1c_ levels between 5.7–6.4% (39–46 mmol/mol) or impaired FPG levels between 5.6–6.9 mmol/l. Type 2 diabetes was defined as HbA_1c_ ≥6.5% (≥48 mmol/mol) or FPG ≥7.0 mmol/l [2]. As internationally recognised risk cut-points for the examined biomarkers have not been established, we classified inflammation and raised immune activation as a level above the study population median for each biomarker (C3, CRP, IL-6, TNF-α, leptin, resistin, PAI-1 and WBC) with the exception of adiponectin (below median level).

### Statistical analysis

Descriptive characteristics were examined according to diagnosis of pre-diabetes and type 2 diabetes. Categorical features are presented as percentages and continuous variables are displayed as a mean (plus or minus one standard deviation) or a median and interquartile range for skewed data. Binary logistic regression was used to explore diabetes-related risk factor and inflammatory biomarker relationships with pre-diabetes (compared to normoglycaemic subjects) and type 2 diabetes (compared to individuals without diabetes) defined using HbA_1c_ and FPG diagnostic cut-points. Models examining metabolic feature associations with pre-diabetes excluded patients with type 2 diabetes indicated by either HbA_1c_ or FPG, a physician diagnosis or Rx diabetes medication use. Risk feature relationships with pre-diabetes (either HbA_1c_ alone, FPG alone or dual categorisation by both HbA_1c_ and FPG) were further evaluated using multinomial logistic regression. Subjects classified as normoglycaemic by both assays were used as the reference category.

The ability of HbA_1c_ to discriminate pre-diabetes (defined by impaired FPG) and type 2 diabetes (defined by FPG levels ≥7.0 mmol/l) was assessed using receiver operating characteristic curve (ROC) analysis. The area under the curve (AUC) provides a scale from 0.5 to 1.0 (with 0.5 representing random chance and 1.0 indicating perfect discrimination) by which to compare the ability of a marker to detect a positive result [18]. The diagnostic properties of different HbA_1c_ thresholds were contrasted by determining sensitivity and false positive rates (FPR). Levels of agreement between diagnostic methods were ascertained using Cohen’s kappa coefficient (K).

Primary data analysis was conducted using IBM SPSS Statistics Version 20 (IBM Corp., Armonk, NY, USA) for Windows. Confidence intervals for prevalence proportions were calculated using the VasserStats statistical website [19]. For all analyses, a P value (two-tailed) of less than 0.05 was considered to indicate statistical significance. Assay results for HbA_1c_ and FPG were available for 1,995 (97.5%) and 1,994 (97.4%) subjects. Participants missing either HbA_1c_ or FPG data were excluded from multinomial and ROC analyses. Low-level missing values were found within most independent variables. Sensitivity analysis indicated a similar percentage of missing data according to either HbA_1c_ or FPG pre-diabetes and diabetes classifications. Missing independent variable data were thus assumed to be ignorable and missing at random.

## Results

### Descriptive characteristics

Characteristics of the study population according to pre-diabetes and type 2 diabetes classifications are presented in Table 1. The prevalence of pre-diabetes was 49.1% (95% CI: 46.9%-51.3%) by elevated HbA_1c_ and 11.5% (95% CI: 10.2%-13.0%) by impaired FPG. Subjects categorised as pre-diabetic using HbA_1c_ had lower BMI and WC levels, lower triglyceride and insulin concentrations, higher HDL-C levels, were less hypertensive, and a greater proportion were female when compared to individuals with pre-diabetes defined by FPG.

**Table 1:** Characteristics of the study population according to pre-diabetes and type 2 diabetes status.

Feature	Full cohort	Pre-diabetes^1^		Type 2 diabetes^2^	
		HbA_1c_	FPG	HbA_1c_	FPG
	(N = 2047)	(N = 980)	(N = 230)	(N = 146)	(N = 85)
Male	1008 (49.2)	441 (45.0)	150 (65.2)	95 (65.1)	59 (69.4)
Age	59.0 (55.0–64.0)	60.0 (55.0–64.0)	61.0 (56.0–65.0)	60.0 (57.0–65.0)	61.0 (56.5–64.5)
Age ≥60	981 (47.9)	510 (52.0)	125 (54.3)	83 (56.8)	51 (60.0)
Diagnosed diabetes	101 (4.9)	-	-	73 (50.0)	51 (60.0)
On Rx for diabetes	78 (3.8)	-	-	60 (41.1)	41 (48.2)
On Rx for hypertension	584 (28.5)	307 (31.3)	98 (42.6)	81 (55.5)	48 (56.5)
On Rx for cholesterol	711 (34.7)	385 (39.3)	93 (40.4)	88 (60.3)	49 (57.6)
BMI (kg/m2)	28.60 ± 4.7	28.80 ± 4.7	30.45 ± 5.2	32.17 ± 5.5	31.81 ± 5.5
BMI ≥30	668 (32.7)	345 (35.2)	109 (47.4)	85 (58.2)	49 (57.6)
WC (cm)	97.04 ± 13.2	97.08 ± 12.9	102.44 ± 12.8	107.91 ± 13.7	108.52 ± 13.9
WC (HIGH)	1119 (54.8)	562 (57.4)	150 (65.2)	119 (81.5)	66 (77.6)
Family diabetes history	390 (19.1)	176 (18.0)	46 (20.0)	62 (42.5)	41 (48.2)
Triglycerides (mmol/l)	1.22 (0.9–1.7)	1.23 (0.9–1.7)	1.41 (1.0–2.0)	1.58 (1.2–2.3)	1.68 (1.2–2.3)
Triglycerides ≥1.7	490 (24.6)	230 (23.8)	85 (37.9)	65 (45.5)	40 (48.8)
HDL-C (mmol/l)	1.45 ± 0.4	1.45 ± 0.4	1.32 ± 0.3	1.17 ± 0.3	1.17 ± 0.4
HDL-C (LOW)	353 (17.6)	165 (17.0)	59 (26.1)	66 (45.2)	35 (41.2)
Dyslipidaemia	168 (8.4)	78 (8.0)	32 (14.0)	37 (25.3)	22 (25.9)
Systolic BP (mmHg)	129.60 ± 16.8	130.10 ± 16.1	134.78 ± 15.5	134.19 ± 17.3	136.24 ± 17.4
Diastolic BP (mmHg)	80.12 ± 9.7	80.24 ± 9.6	82.25 ± 9.1	79.50 ± 10.3	80.72 ± 10.5
BP ≥130/85	1045 (51.3)	521 (53.4)	155 (67.7)	89 (61.4)	56 (66.7)
HbA1c (%)	5.7 (5.5–6.0)	5.9 (5.7–6.0)	5.8 (5.6–6.1)	7.0 (6.7–8.1)	7.6 (6.8–9.0)
HbA1c (mmol/mol)	39 (37–42)	41 (39–42)	40 (38–43)	53 (50–65)	60 (51–75)
FPG (mmol/l)	4.90 (4.7–5.4)	5.00 (4.7–5.3)	5.80 (5.7–6.1)	6.90 (6.0–9.0)	8.50 (7.6–10.8)
Insulin (μU/ml)	8.65 (5.3–14.1)	8.98 (4.6–11.8)	12.67 (7.4–19.5)	18.27 (10.6–31.9)	19.21 (12.1–30.9)
Insulin 75th percentile	497 (25.0)	238 (24.6)	98 (43.2)	94 (65.7)	59 (70.2)
≥3 MetS features^3^	606 (29.6)	298 (30.4)	112 (48.7)	103 (70.5)	63 (74.1)
C3 (mg/dl)	135.92 ± 24.7	138.85 ± 24.5	141.41 ± 25.8	148.13 ± 28.6	149.20 ± 24.9
CRP (ng/ml)	1.35 (1.0–2.3)	1.43 (1.0–2.4)	1.38 (1.0–2.3)	1.79 (1.1–3.2)	1.91 (1.2–3.0)
IL-6 (pg/ml)	1.81 (1.2–2.9)	1.91 (1.3–3.0)	2.02 (1.5–3.0)	2.92 (1.7–4.8)	2.83 (1.8–4.6)
TNF-α (pg/ml)	5.97 (4.9–7.3)	6.02 (5.0–7.3)	5.94 (4.9–7.5)	6.99 (5.5–8.3)	7.09 (5.6–8.1)
Adiponectin (ng/ml)	4.75 (2.9–7.5)	4.92 (3.1–7.5)	3.63 (2.4–5.6)	2.82 (1.7–4.6)	2.73 (1.9–4.7)
Leptin (ng/ml)	1.95 (1.1–3.1)	2.09 (1.3–3.5)	2.06 (1.3–3.8)	2.28 (1.3–3.9)	2.09 (1.1–3.4)
Resistin (ng/ml)	5.07 (3.9–6.7)	4.93 (3.8–6.6)	4.89 (3.7–6.7)	6.15 (4.6–7.3)	5.53 (4.5–7.3)
PAI-1 (ng/ml)	27.38 ± 12.6	27.87 ± 12.0	29.56 ± 13.2	31.35 ± 15.9	30.03 ± 11.0
WBC (109/l)	6.00 ± 1.9	6.12 ± 2.1	6.33 ± 1.72	7.39 ± 2.4	7.21 ± 1.9

Mean and ± standard deviation are shown for continuous variables. Age, triglycerides, HbA_1c_, FPG, insulin, CRP, IL-6, TNF-α, adiponectin, leptin and resistin are shown as a median (interquartile range). Numbers and % (in brackets) for categorical variables will vary in different analyses as some variables have missing values.

^1^Pre-diabetes: HbA_1c_ levels 5.7–6.4% (39–46 mmol/mol) or FPG levels 5.6–6.9 mmol/l.

^2^Type 2 diabetes: HbA_1c_ ≥6.5% (≥48 mmol/mol) or FPG ≥7.0 mmol/l.

^3^MetS features: WC (HIGH), triglycerides ≥1.7, HDL-C (LOW), BP ≥130/85 or Rx and insulin 75^th^ percentile.

### Logistic regression

In binary logistic regression analyses (Table 2), associations between commonly assessed diabetes risk factors and pre-diabetes were stronger in subjects diagnosed by FPG. Odds ratios for pre-diabetes indicated by HbA_1c_ were non-significant for having a family diabetes history and elevated triglyceride levels, while there was a three-fold increased likelihood (OR: 3.0, 95% CI: 2.2–3.9) of having ≥3 MetS features in participants identified by FPG compared to an odds ratio of 1.6 (95% CI: 1.3–2.0) in pre-diabetes by HbA_1c_. In contrast, metabolic risk factor relationships with type 2 diabetes were generally comparable according to diagnosis by either assay, with odds ratios of having ≥3 MetS features being 6.1 (95% CI: 4.2–8.8) and 6.8 (95% CI: 4.1–11.2) for subjects diagnosed by HbA_1c_ and FPG respectively. Regardless of definition, patients with pre-diabetes and type 2 diabetes displayed a chronic pro-inflammatory profile as characterised by elevated C3, IL-6, WBC levels and reduced adiponectin concentrations.

**Table 2:** Odds ratios (95% CI) of having risk factors according to diagnosis of pre-diabetes and type 2 diabetes by HbA_1c_ or FPG.

Feature	Odds ratios (95% CI)^1^							
	Pre-diabetes compared to normoglycaemia^2^				Type 2 diabetes compared to no diabetes^3^			
	HbA_1c_	P value	FPG	P value	HbA_1c_	P value	FPG	P value
Male	0.8 (0.6–0.9)	<0.001	2.3 (1.7–3.0)	<0.001	2.0 (1.4–2.9)	<0.001	2.5 (1.5–3.9)	<0.001
Age ≥60	1.6 (1.3–1.9)	<0.001	1.4 (1.1–1.9)	0.011	1.5 (1.1–2.2)	0.018	1.7 (1.1–2.7)	0.017
Family diabetes history	1.2 (0.9–1.5)	0.182	1.4 (1.0–2.1)	0.043	4.1 (2.9–5.9)	<0.001	5.2 (3.3–8.1)	<0.001
BMI ≥30	1.8 (1.4–2.2)	<0.001	2.2 (1.7–3.0)	<0.001	3.1 (2.2–4.3)	<0.001	2.8 (1.8–4.4)	<0.001
WC (HIGH)	1.5 (1.2–1.9)	0.001	2.0 (1.4–3.1)	0.001	5.4 (2.5–11.8)	<0.001	7.4 (2.3–23.5)	0.001
Triglycerides ≥1.7	1.2 (0.9–1.5)	0.134	2.1 (1.5–2.8)	<0.001	2.5 (1.8–3.6)	<0.001	2.8 (1.8–4.4)	<0.001
HDL-C (LOW)	1.4 (1.1–1.8)	0.018	2.3 (1.7–3.3)	<0.001	4.6 (3.2–6.6)	<0.001	3.6 (2.3–5.7)	<0.001
Dyslipidaemia	1.6 (1.1–2.4)	0.019	2.6 (1.7–4.1)	<0.001	4.3 (2.8–6.5)	<0.001	4.1 (2.4–6.9)	<0.001
BP ≥130/85 or Rx	1.4 (1.2–1.7)	<0.001	2.5 (1.8–3.5)	<0.001	3.0 (1.9–4.8)	<0.001	4.4 (2.2–8.6)	<0.001
Insulin 75^th^ percentile	1.6 (1.3–2.0)	<0.001	3.1 (2.3–4.2)	<0.001	6.5 (4.5–9.4)	<0.001	7.2 (4.4–11.7)	<0.001
≥3 MetS features^4^	1.6 (1.3–2.0)	<0.001	3.0 (2.2–3.9)	<0.001	6.1 (4.2–8.8)	<0.001	6.8 (4.1–11.2)	<0.001
C3^5^	1.8 (1.5–2.2)	<0.001	1.4 (1.0–1.8)	0.032	3.3 (2.2–4.9)	<0.001	3.1 (1.9–5.0)	<0.001
CRP^5^	1.4 (1.1–1.7)	0.001	1.2 (0.9–1.5)	0.293	1.5 (1.1–2.2)	0.02	1.6 (1.0–2.6)	0.032
IL-6^5^	1.6 (1.3–1.9)	<0.001	1.5 (1.1–2.0)	0.005	2.8 (1.9–4.1)	<0.001	2.8 (1.7–4.6)	<0.001
TNF-α^5^	1.2 (1.0–1.4)	0.078	1.0 (0.7–1.3)	0.738	2.3 (1.6–3.3)	<0.001	2.7 (1.6–4.4)	<0.001
Adiponectin^5^	1.4 (1.1–1.7)	0.004	2.0 (1.4–2.7)	<0.001	4.0 (2.5–6.2)	<0.001	3.2 (1.8–5.6)	<0.001
Leptin^5^	1.5 (1.2–1.8)	<0.001	1.4 (1.1–1.9)	0.014	1.5 (1.0–2.1)	0.026	1.2 (0.8–1.8)	0.48
Resistin^5^	0.9 (0.8–1.1)	0.305	0.9 (0.7–1.2)	0.391	2.4 (1.7–3.5)	<0.001	1.8 (1.1–2.8)	0.012
PAI-1^5^	1.3 (1.1–1.6)	0.005	1.3 (1.0–1.7)	0.108	1.5 (1.0–2.1)	0.028	1.5 (1.0–2.4)	0.078
WBC^5^	1.7 (1.4–2.1)	<0.001	1.6 (1.2–2.1)	0.001	3.4 (2.3–5.0)	<0.001	3.3 (2.0–5.5)	<0.001

^1^Binary logistic regression. Gender adjusted for age (continuous), age ≥60 adjusted for gender, all other variables adjusted for age (continuous) and gender.

^2^Pre-diabetes: HbA_1c_ ≥5.7% (≥39 mmol/mol) or FPG ≥5.6 mmol/l, models exclude subjects with type 2 diabetes: HbA_1c_ ≥6.5% (≥48 mmol/mol) or FPG ≥7.0 mmol/l or physician diagnosis or Rx diabetes medication use.

^3^Models exclude 24 subjects that indicated a physician diagnosis or Rx diabetes medication use but who did not have positive HbA_1c_ or FPG test results.

^4^MetS features: WC (HIGH), triglycerides ≥1.7, HDL-C (LOW), BP ≥130/85 or Rx and insulin 75^th^ percentile.

^5^Threshold: above median level in the study population except adiponectin (below median level).

The results from multinomial regression models exploring risk factor relationships with pre-diabetes classified by HbA_1c_ alone, FPG alone, or by both HbA_1c_ and FPG together are displayed in Table 3. Odds ratios for obesity, elevated BP, increased insulin concentrations and MetS were higher in participants classified by both assays, with four-fold increased odds (OR: 4.0, 95% CI: 2.8–5.8) of having ≥3 MetS features, compared to either HbA_1c_ (OR: 1.4, 95% CI: 1.2–1.8) or FPG (OR: 3.0, 95% CI: 1.7–5.1) alone. Stronger associations with markers of inflammation were also observed in subjects identified by both criteria.

**Table 3:** Odds ratios (95% CI) of having risk factors according to diagnosis of pre-diabetes^1^ by HbA_1c_ alone, FPG alone, or by both HbA_1c_ and FPG together.

Feature	Odds ratios (95% CI)^2^					
	HbA_1c_ alone	P value	FPG alone	P value	HbA_1c_ & FPG	P value
	(N = 814)		(N = 62)		(N = 162)	
Male	0.8 (0.6–0.9)	0.006	3.3 (1.8–5.9)	<0.001	1.6 (1.2–2.3)	0.005
Age ≥60	1.6 (1.3–1.9)	<0.001	1.4 (0.8–2.3)	0.251	2.0 (1.4–2.8)	<0.001
Family diabetes history	1.1 (0.8–1.4)	0.474	1.2 (0.6–2.4)	0.651	1.7 (1.1–2.6)	0.013
BMI ≥30	1.6 (1.3–2.0)	<0.001	1.7 (1.0–3.0)	0.051	3.4 (2.4–4.9)	<0.001
WC (HIGH)	1.4 (1.2–1.8)	<0.001	2.0 (1.2–3.4)	0.011	2.6 (1.8–3.7)	<0.001
Triglycerides ≥1.7	1.2 (0.9–1.5)	0.267	2.5 (1.4–4.3)	0.001	2.3 (1.4–4.3)	<0.001
HDL-C (LOW)	1.3 (1.0–1.7)	0.095	2.5 (1.3–4.7)	0.004	2.8 (1.8–4.3)	<0.001
Dyslipidaemia	1.6 (1.0–2.5)	0.041	3.5 (1.6–7.8)	0.002	3.5 (2.0–6.2)	<0.001
BP ≥130/85 or Rx	1.3 (1.0–1.6)	0.012	2.2 (1.2–3.9)	0.009	3.3 (2.2–5.1)	<0.001
Insulin 75^th^ percentile	1.5 (1.2–2.0)	0.002	3.4 (2.0–5.9)	<0.001	4.1 (2.8–5.9)	<0.001
≥3 MetS features^3^	1.4 (1.2–1.8)	0.003	3.0 (1.7–5.1)	<0.001	4.0 (2.8–5.8)	<0.001
C3^4^	1.8 (1.5–2.3)	<0.001	1.4 (0.9–2.4)	0.17	2.2 (1.5–3.1)	<0.001
CRP^4^	1.4 (1.1–1.7)	0.002	1.1 (0.7–2.0)	0.640	1.5 (1.1–2.2)	0.017
IL-6^4^	1.5 (1.2–1.9)	<0.001	1.4 (0.8–2.4)	0.212	2.1 (1.5–3.0)	<0.001
TNF-α^4^	1.2 (1.0–1.5)	0.096	0.8 (0.5–1.4)	0.524	1.1 (0.8–1.6)	0.446
Adiponectin^4^	1.3 (1.0–1.6)	0.043	1.3 (0.7–2.3)	0.373	2.6 (1.8–3.9)	<0.001
Leptin^4^	1.4 (1.2–1.8)	<0.001	1.3 (0.8–2.2)	0.345	2.0 (1.4–2.9)	<0.001
Resistin^4^	1.0 (0.8–1.2)	0.626	1.3 (0.7–2.1)	0.389	0.8 (0.5–1.1)	0.139
PAI-1^4^	1.3 (1.1–1.6)	0.008	1.4 (0.8–2.4)	0.2	1.6 (1.1–2.2)	0.014
WBC^4^	1.6 (1.3–2.0)	<0.001	1.3 (0.7–2.2)	0.371	2.6 (1.8–3.7)	<0.001

^1^Pre-diabetes: HbA_1c_ ≥5.7% (≥39 mmol/mol) or FPG ≥5.6 mmol/l, models exclude subjects with type 2 diabetes: HbA_1c_ ≥6.5% (≥48 mmol/mol) or FPG ≥7.0 mmol/l or physician diagnosis or Rx diabetes medication use.

^2^Multinomial logistic regression, reference category: normoglycaemia by both HbA_1c_ and FPG. Gender adjusted for age (continuous), age ≥60 adjusted for gender, all other variables adjusted for age (continuous) and gender.

^3^MetS features: WC (HIGH), triglycerides ≥1.7, HDL-C (LOW), BP ≥130/85 or Rx and insulin 75^th^ percentile.

^4^Threshold: above median level in the study population except adiponectin (below median level).

### ROC analysis

**Fig 1:**
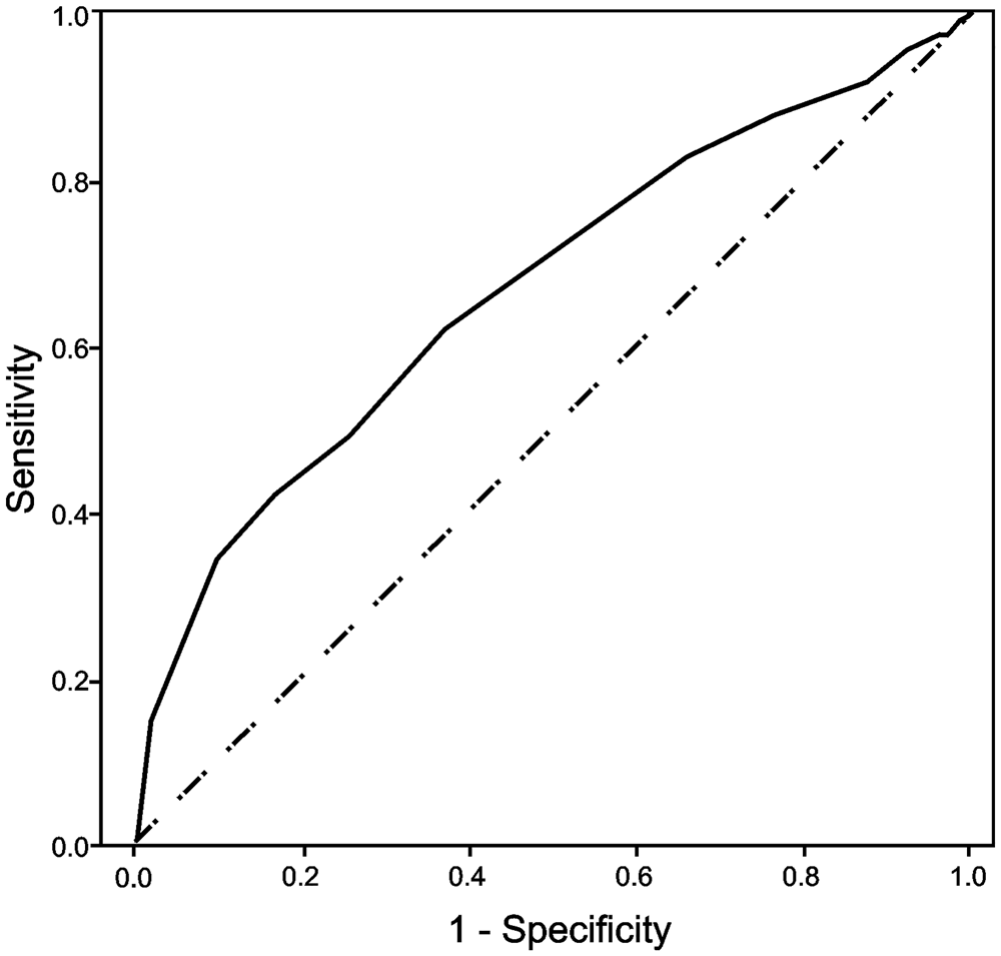
Receiver operating characteristic curve for HbA_1c_ to discriminate subjects with pre-diabetes. The figure shows an ROC curve for HbA_1c_ (continuous) to discriminate subjects with pre-diabetes (impaired FPG ≥5.6 mmol/l). The area under the curve value was AUC: 0.668, (95% CI: 0.627–0.710).

**Fig 2:**
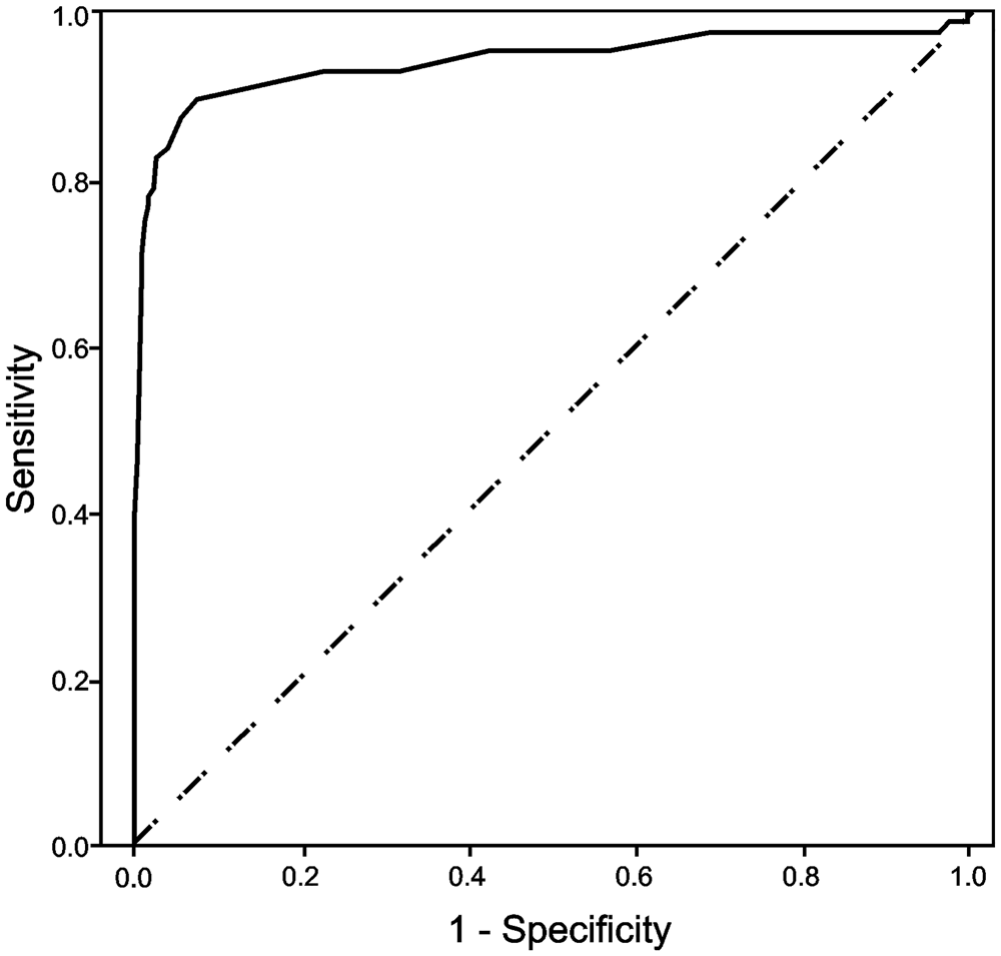
Receiver operating characteristic curve for HbA_1c_ to discriminate subjects with type 2 diabetes. The figure shows an ROC curve for HbA_1c_ (continuous) to discriminate subjects with type 2 diabetes (FPG ≥7.0 mmol/l). The area under the curve value was AUC: 0.941, (95% CI: 0.902–0.980).

Receiver operating characteristic curves for HbA_1c_ to detect pre-diabetes and type 2 diabetes are shown in Figs 1 and 2. The ability of HbA_1c_ to discriminate pre-diabetes characterised by impaired FPG was low (AUC: 0.668, 95% CI: 0.627–0.710). The HbA_1c_ ≥5.7% (≥39 mmol/mol) pre-diabetes threshold demonstrated marginal sensitivity (72%) and a high FPR (52%). The level of agreement between both diagnostic methods was also poor (K: 0.084). Discriminatory capacity for type 2 diabetes defined by FPG ≥7.0 mmol/l was high (AUC: 0.941, 95% CI: 0.902–0.980). Sensitivity, FPR and kappa for the ADA-recommended HbA_1c_ ≥6.5% (≥48 mmol/mol) cut-off were 84%, 4% and 0.60 respectively.

## Discussion

In this study of 2,047 middle-aged Caucasian-European men and women we show that subjects with HbA_1c_ levels 5.7–6.4% (39–46 mmol/mol) or FPG levels 5.6–6.9 mmol/l may exhibit different cardiometabolic profiles. Stronger relationships with diabetes-related risk features were found using impaired FPG compared to elevated HbA_1c_ to diagnose pre-diabetes. Conversely, the metabolic profiles of patients with type 2 diabetes, defined by either HbA_1c_ ≥6.5% (≥48 mmol/mol) or FPG ≥7.0 mmol/l concentrations, were broadly similar. In addition, it was noted that associations with risk factors and inflammatory markers were higher in pre-diabetic individuals classified by both assays. These results suggest that a combination of both criteria may be useful for detecting subjects at increased cardiometabolic risk.

Noticeably, within this population, a higher percentage of patients were identified as having pre-diabetes by HbA_1c_ (49.1% vs. 11.5% for FPG). A higher prevalence of pre-diabetes by HbA_1c_ in a United Kingdom cohort (N = 8,696) was also noted by Mostafa et al. [20], who reported a prevalence of 44.9% in participants diagnosed by HbA_1c_ compared to 16.2% in subjects detected by an oral glucose tolerance test (OGTT). Similar findings were determined using FPG as the glucose-based criterion. Our results are also consistent with those reported in a recent Chinese study (N = 2,318) and from research examining a Palestinian Arab population (N = 1,370). Du et al. [21] and Kharroubi et al. [22] found reasonable or moderate concordance between HbA_1c_ and FPG for type 2 diabetes, but a higher prevalence by HbA_1c_ and limited overlap for pre-diabetes using ADA-designated thresholds.

However, our results contrast with findings reported in the United States by the Insulin Resistance Atherosclerosis Study (N = 855), which found a higher prevalence of pre-diabetes by FPG (31.1% vs. 10.6% for HbA_1c_) [23]. Similarly, research utilising data from the National Health and Nutrition Examination Survey (1999–2006) found the prevalence of pre-diabetes in a sample of 7,029 adults to be 28.2% and 12.6% using FPG and HbA_1c_ respectively [24]. Possible reasons for observed prevalence disparities between HbA_1c_ and FPG may include age, gender or ethnic differences in examined populations [10, 14, 15]. In addition, as glucose continues to be metabolized in blood cells even after sampling, discrepancies may be due to biochemical analysis intervals within different studies [7, 22].

Longitudinal research has suggested that combined use of HbA_1c_ and FPG may be beneficial for identifying high-risk subjects. In two Asian studies, Inoue et al. [25] and Heianza et al. [26] demonstrated hazard ratios for type 2 diabetes to be greater for subjects classified by both assays when compared to those diagnosed by either HbA_1c_ or FPG separately. Findings from the Kansai Healthcare Study showed that joint use of both methods improved predictive ability [27]. In ROC analysis, AUCs for models including both HbA_1c_ and FPG were larger than those for HbA_1c_ (0.853 vs. 0.771; P<0.001) or FPG (0.853 vs. 0.818; P<0.001) alone. Recent research by Lipska et al. also revealed that addition of HbA_1c_ to a model with impaired FPG improved discrimination and calibration [28]. The results from the present study imply that the mechanism for this association is that individuals with diabetes-related phenotypes are more accurately identified using combined criteria.

Established risk factors for type 2 diabetes include obesity, raised triglyceride and low HDL-C levels, hypertension and insulin resistance [29]. In particular, subjects with a combination of these features have been shown to have a five-fold increased risk of developing diabetes [30]. Cardiovascular diseases, and in particular obesity-related type 2 diabetes, are also characterised by a low-grade but chronic inflammatory state [31, 32]. This may be reflected in an increased production of pro-inflammatory cytokines and also in higher levels of acute-phase response proteins, coagulation factors, macrophages and immune cells and lower levels of adiponectin, the anti-inflammatory adipokine [32, 33].

In our study it was noted that pre-diabetic individuals categorised by both assays demonstrated a stronger association with cardiometabolic feature clustering and displayed a more pro-inflammatory, pro-atherogenic, hypertensive and insulin resistant profile. Though few prospective studies have comprehensively identified features related to pre-diabetes development, it has been suggested that risk factors for pre-diabetes mirror those for type 2 diabetes [34]. Consequently, on the basis of the similar risk profiles noted in this study between pre-diabetes (defined using both HbA_1c_ and FPG) and type 2 diabetes (classified by either method), these findings also indicate that combined use of both assays may be clinically useful for detecting individuals at highest odds of developing diabetes.

Although HbA_1c_ has long been used as a marker for glycaemic control, its diagnostic performance for type 2 diabetes is still questioned [35–37]. Though a more expensive assay, when compared with FPG, HbA_1c_ has advantages including convenience, greater pre-analytical stability, lower biological variability and increasing international standardisation [7, 37]. Moreover, HbA_1c_ has been shown to correlate with cardiovascular disease and all-cause mortality [38]. However, as diabetes is clinically defined by elevated blood glucose, and not by glycation of proteins, there is concern that using HbA_1c_ to classify type 2 diabetes may lead to major changes in the pathophysiological paradigm that defines the condition [7]. Although a report from the United States inferred that diagnosis by HbA_1c_, rather than FPG, would not significantly alter type 2 diabetes prevalence, and that categorisation would remain unchanged in as many as 97.7% of subjects [39], evidence is still equivocal [40].

Notably, within our sample, a higher prevalence of diabetes was determined using HbA_1c_ (7.3%, 95% CI: 6.3%-8.6%) compared to FPG (4.3%, 95% CI: 3.5%-5.2%). However, a similar type 2 diabetes prevalence rate in middle-aged Irish adults, defined by HbA_1c_, was recently reported using data from the nationally representative 2007 Survey of Lifestyle, Attitudes and Nutrition (7.1%, 95% CI: 5.2%-9.0%) [40, 41]. It was also noted that diabetic subjects identified by HbA_1c_ or FPG within the present study displayed markedly similar cardiometabolic profiles. In addition, HbA_1c_ demonstrated high predictive ability for type 2 diabetes diagnosed by FPG ≥7.0 mmol/l levels. Conversely, HbA_1c_ showed poor discriminatory capacity for pre-diabetes defined by impaired FPG.

As HbA_1c_ reflects long-term glycaemic exposure, including postprandial glucose spikes, rather than the acute dysglycaemia indicated by FPG, it is rational to assume that each assay may identify different individuals. Our results suggest that HbA_1c_ may provide greater sensitivity for diagnosing type 2 diabetes within this sample. However, the limited overlap and substantially varied cardiometabolic profiles in subjects diagnosed with pre-diabetes, by either HbA_1c_ or FPG, imply that HbA_1c_ alone may lack specificity to accurately detect individuals at risk of diabetes development. It was also noted that metabolic risk profiles in pre-diabetic subjects, classified by impaired FPG levels only, were also considerably increased. This indicates that a percentage of high-risk individuals would be missed if HbA_1c_ was employed as a sole diagnostic criterion.

This study has several strengths, including a high participation rate (67%). As far as we are aware, ours is the first to compare pre-diabetes and type 2 diabetes prevalence, defined using both HbA_1c_ and FPG criteria, in a middle-aged Irish population. Additionally, few studies have compared a broad range of metabolic risk features and biomarkers with pre-diabetes and type 2 diabetes diagnosed by both assays. Our results are of potential clinical significance in terms of screening and the use of HbA_1c_ as a method for diagnosing diabetes and determining cardiometabolic risk. Accurate estimates of progression rates to type 2 diabetes are needed for efficient allocation of resources and to optimise public health prevention strategies [42]. Importantly, our findings indicate that caution should be taken with regard to how risk is defined as inexact methods may overestimate future diabetes burden [43, 44].

Notwithstanding these strengths, several limitations can be identified. These include single measurements of HbA_1c_ and FPG and that we did not have OGTT results as a comparison test. Although use of a third assay would have allowed a more thorough evaluation of HbA_1c_ and FPG, as discussed by Bonora et al. [7] comparisons between diagnostic methods for pre-diabetes and type 2 diabetes are ambiguous, as a true gold standard test is unavailable. Also, cross-sectional data precludes examination of temporal relationships. Consequently, though results from our research suggest associations between variables, they do not demonstrate an ability to predict type 2 diabetes or future cardiovascular events.

Equally of concern is that our data were derived from a single primary care based sample. Although results from the Cork and Kerry Diabetes and Heart Disease Study demonstrate prevalence rates for obesity and cardiovascular outcomes similar to those observed in other nationally representative Irish studies [40, 41, 45], the possibility that this sample is not representative of the source population must be acknowledged. However, previous research suggests that approximately 98% of Irish adults are registered with a GP and that, even in the absence of a universal patient registration system, it is possible to perform population-based epidemiological studies that are representative using these methods [46]. In addition, Ireland presents a generally ethnically homogeneous population [47]. Thus, the associations we observed between cardiometabolic features and HbA_1c_ and FPG may be comparable to other middle-aged Irish adults. As random sampling of subjects and the use of validated methods for data collection ensured internal sample validity, it is equally possible that the relationships described may be generalisable to a similar middle-aged, Caucasian-European population. Nevertheless, future studies utilising longitudinal data in different samples will be needed to confirm these findings. In particular, it will be necessary to determine whether risk stratification, using both assays, is clinically useful as a method for predicting type 2 diabetes.

## Conclusions

In summary, our results suggest that in middle-aged Caucasian-Europeans, when using ADA-recommended cut-points, HbA_1c_ alone is a poor indicator of diabetes risk, but is appropriate for type 2 diabetes diagnosis. Furthermore, combined use of HbA_1c_ and FPG identifies subjects at substantially increased cardiometabolic risk. Although the efficacy and cost-effectiveness of routine screening for diabetes in primary care has not been established [48–50], in light of the increasing prevalence of type 2 diabetes worldwide, there is a need to identify high-risk subjects. Dual screening, utilising both HbA_1c_ and FPG, may provide a more accurate method for predicting cardiometabolic events. Earlier diagnosis could enable earlier targeted interventions or therapies, thus attenuating development of type 2 diabetes and associated cardiovascular complications.

## Supporting Information

S1 File The Cork and Kerry Diabetes and Heart Disease Study (Phase II) Dataset. (ZIP)

## References

[1] Wild S, Roglic G, Green A, Sicree R, King H. Global prevalence of diabetes estimates for the year 2000 and projections for 2030. Diabetes Care. 2004;27(5):1047–53. 15111519 10.2337/diacare.27.5.1047

[2] Association AD. Diagnosis and classification of diabetes mellitus. Diabetes Care. 2013;36(Suppl 1):S67–S74. 10.2337/dc13-S067 23264425 PMC3537273

[3] Calle M, Fernandez M. Inflammation and type 2 diabetes. Diabetes & metabolism. 2012;38(3):183–91. 22252015 10.1016/j.diabet.2011.11.006

[4] Grundy SM, Benjamin IJ, Burke GL, Chait A, Eckel RH, Howard BV, et al Diabetes and cardiovascular disease a statement for healthcare professionals from the American Heart Association. Circulation. 1999;100(10):1134–46. 10477542 10.1161/01.cir.100.10.1134

[5] Tabak AG, Herder C, Rathmann W, Brunner EJ, Kivimäki M. Prediabetes: a high-risk state for diabetes development. The Lancet. 2012;379(9833):2279–90. 10.1016/S0140-6736(12)60283-9 PMC3891203 22683128

[6] Gillett MJ. International Expert Committee Report on the Role of the A1C Assay in the Diagnosis of Diabetes: Diabetes Care 2009; 32 (7): 1327–1334. The Clinical Biochemist Reviews. 2009;30(4):197. 20011212 PMC2791773

[7] Bonora E, Tuomilehto J. The pros and cons of diagnosing diabetes with A1C. Diabetes Care. 2011;34(Supplement 2):S184–S90. 21525453 10.2337/dc11-s216 PMC3632159

[8] Church D, Simmons D. More evidence of the problems of using HbA1c for diagnosing diabetes? The known knowns, the known unknowns and the unknown unknowns. Journal of internal medicine. 2014;276(2):171–3. 10.1111/joim.12200 24443985

[9] Saukkonen T, Cederberg H, Jokelainen J, Laakso M, Härkönen P, Keinänen-Kiukaanniemi S, et al Limited Overlap Between Intermediate Hyperglycemia as Defined by A1C 5.7–6.4%, Impaired Fasting Glucose, and Impaired Glucose Tolerance. Diabetes Care. 2011;34(10):2314–6. 10.2337/dc11-0183 21816975 PMC3177731

[10] Kim JH, Shin JH, Lee HJ, Kim SY, Bae HY. Discordance between HbA1c and fasting plasma glucose criteria for diabetes screening is associated with obesity and old age in Korean individuals. Diabetes research and clinical practice. 2011;94(2):e27–e9. 10.1016/j.diabres.2011.07.013 21835487

[11] Marini MA, Succurro E, Castaldo E, Cufone S, Arturi F, Sciacqua A, et al Cardiometabolic risk profiles and carotid atherosclerosis in individuals with prediabetes identified by fasting glucose, postchallenge glucose, and hemoglobin A1c criteria. Diabetes Care. 2012;35(5):1144–9. 10.2337/dc11-2032 22399698 PMC3329850

[12] Rathmann W, Kowall B, Tamayo T, Giani G, Holle R, Thorand B, et al Hemoglobin A1c and glucose criteria identify different subjects as having type 2 diabetes in middle-aged and older populations: The KORA S4/F4 Study. Annals of Medicine. 2012;44(2):170–7. 10.3109/07853890.2010.531759 21091229

[13] Lipska KJ, De Rekeneire N, Van Ness PH, Johnson KC, Kanaya A, Koster A, et al Identifying dysglycemic states in older adults: implications of the emerging use of hemoglobin A1c. Journal of Clinical Endocrinology & Metabolism. 2010;95(12):5289–95. 20861123 10.1210/jc.2010-1171 PMC2999974

[14] Inoue M, Inoue K, Akimoto K. Effects of Age and Sex in the Diagnosis of Type 2 Diabetes Using Glycated Haemoglobin in Japan: The Yuport Medical Checkup Centre Study. PloS one. 2012;7(7):e40375 10.1371/journal.pone.0040375 22792294 PMC3390388

[15] Wolffenbuttel BHR, Herman WH, Gross JL, Dharmalingam M, Honghua H J, Hardin DS. Ethnic Differences in Glycemic Markers in Patients With Type 2 Diabetes. Diabetes Care. 2013;36(10):2931–6. 10.2337/dc12-2711 . 23757434 PMC3781497

[16] Kearney PM, Harrington JM, Mc Carthy VJ, Fitzgerald AP, Perry IJ. Cohort Profile: The Cork and Kerry Diabetes and Heart Disease Study. Int J Epidemiol. 2013;42(5):1253–62. Epub 2012/09/18. 10.1093/ije/dys131 . 22984148

[17] Grundy SM, Brewer HB Jr, Cleeman JI, Smith SC Jr, Lenfant C. Definition of metabolic syndrome report of the National Heart, Lung, and Blood Institute/American Heart Association Conference on scientific issues related to definition. Circulation. 2004;109(3):433–8. 14744958 10.1161/01.CIR.0000111245.75752.C6

[18] Peat J, Barton B. Medical statistics: A guide to data analysis and critical appraisal: BMJ Books; 2008.

[19] Lowry R. VassarStats. The confidence interval of a proportion. Available: http://www.vassarstats.net/prop1.html.2012 December 2012. Available from: http://www.vassarstats.net/prop1.html.

[20] Mostafa SA, Khunti K, Srinivasan BT, Webb D, Gray LJ, Davies MJ. The potential impact and optimal cut-points of using glycated haemoglobin, HbA1c, to detect people with impaired glucose regulation in a UK multi-ethnic cohort. Diabetes research and clinical practice. 2010;90(1):100 10.1016/j.diabres.2010.06.008 20633944

[21] Du TT, Yin P, Zhang JH, Zhang D, Shi W, Yu XF. Comparison of the performance of HbA1c and fasting plasma glucose in identifying dysglycaemic status in Chinese high‐risk subjects. Clinical and Experimental Pharmacology and Physiology. 2013;40(2):63–8. 10.1111/1440-1681.12038 23198814

[22] Kharroubi AT, Darwish HM, Al-Halaweh AIA, Khammash UM. Evaluation of glycated hemoglobin (HbA1c) for diagnosing type 2 diabetes and prediabetes among Palestinian Arab population. PloS one. 2014;9(2):e88123 10.1371/journal.pone.0088123 24505401 PMC3914917

[23] Lorenzo C, Wagenknecht LE, Hanley AJ, Rewers MJ, Karter AJ, Haffner SM. A1C Between 5.7 and 6.4% as a Marker for Identifying Pre-Diabetes, Insulin Sensitivity and Secretion, and Cardiovascular Risk Factors The Insulin Resistance Atherosclerosis Study (IRAS). Diabetes Care. 2010;33(9):2104–9. 10.2337/dc10-0679 20573754 PMC2928372

[24] Mann DM, Carson AP, Shimbo D, Fonseca V, Fox CS, Muntner P. Impact of A1C screening criterion on the diagnosis of pre-diabetes among U.S. adults. Diabetes Care. 2010;33(10):2190–5. Epub 2010/07/16. 10.2337/dc10-0752 20628087 PMC2945159

[25] Inoue K, Matsumoto M, Akimoto K. Fasting plasma glucose and HbA1c as risk factors for type 2 diabetes. Diabetic Medicine. 2008;25(10):1157–63. 10.1111/j.1464-5491.2008.02572.x 19046193

[26] Heianza Y, Hara S, Arase Y, Saito K, Fujiwara K, Tsuji H, et al HbA1c 5.7–6.4% and impaired fasting plasma glucose for diagnosis of prediabetes and risk of progression to diabetes in Japan (TOPICS 3): a longitudinal cohort study. Lancet. 2011;378(9786):147–55. Epub 2011/06/28. 10.1016/s0140-6736(11)60472-8 . 21705064

[27] Sato KK, Hayashi T, Harita N, Yoneda T, Nakamura Y, Endo G, et al Combined measurement of fasting plasma glucose and A1C is effective for the prediction of type 2 diabetes the Kansai Healthcare Study. Diabetes Care. 2009;32(4):644–6. 10.2337/dc08-1631 19131461 PMC2660452

[28] Lipska KJ, Inzucchi SE, Van Ness PH, Gill TM, Kanaya A, Strotmeyer ES, et al Elevated HbA1c and Fasting Plasma Glucose in Predicting Diabetes Incidence Among Older Adults Are two better than one? Diabetes Care. 2013;36(12):3923–9. 10.2337/dc12-2631 24135387 PMC3836095

[29] Alberti K, Zimmet P, Shaw J. Metabolic syndrome—a new world‐wide definition. A Consensus Statement from the International Diabetes Federation. Diabetic Medicine. 2006;23(5):469–80. 16681555 10.1111/j.1464-5491.2006.01858.x

[30] Stern MP, Williams K, González-Villalpando C, Hunt KJ, Haffner SM. Does the metabolic syndrome improve identification of individuals at risk of type 2 diabetes and/or cardiovascular disease? Diabetes Care. 2004;27(11):2676–81. 15505004 10.2337/diacare.27.11.2676

[31] Hotamisligil GS. Inflammation and metabolic disorders. Nature. 2006;444(7121):860–7. 17167474 10.1038/nature05485

[32] Phillips CM, Perry IJ. Does Inflammation Determine Metabolic Health Status in Obese and Nonobese Adults? The Journal of Clinical Endocrinology & Metabolism. 2013;98(10):E1610–E9. 23979951 10.1210/jc.2013-2038

[33] Van Greevenbroek M, Schalkwijk C, Stehouwer C. Obesity-associated low-grade inflammation in type 2 diabetes mellitus: causes and consequences. Neth J Med. 2013;71(4):174–87. 23723111

[34] Twigg SM, Kamp MC, Davis TM, Neylon EK, Flack JR. Prediabetes: a position statement from the Australian Diabetes Society and Australian Diabetes Educators Association. Medical journal of Australia. 2007;186(9):461 17484708 10.5694/j.1326-5377.2007.tb00998.x

[35] Lapolla A, Mosca A, Fedele D. The general use of glycated haemoglobin for the diagnosis of diabetes and other categories of glucose intolerance: still a long way to go. Nutrition, Metabolism and Cardiovascular Diseases. 2011;21(7):467–75. 10.1016/j.numecd.2011.02.006 21641782

[36] Olson DE, Rhee MK, Herrick K, Ziemer DC, Twombly JG, Phillips LS. Screening for diabetes and pre-diabetes with proposed A1C-based diagnostic criteria. Diabetes Care. 2010;33(10):2184–9. 10.2337/dc10-0433 20639452 PMC2945158

[37] Cohen RM, Haggerty S, Herman WH. HbA1c for the diagnosis of diabetes and prediabetes: is it time for a mid-course correction? Journal of Clinical Endocrinology & Metabolism. 2010;95(12):5203–6. 21131541 10.1210/jc.2010-2352 PMC2999978

[38] Khaw K-T, Wareham N, Bingham S, Luben R, Welch A, Day N. Association of hemoglobin A1c with cardiovascular disease and mortality in adults: the European prospective investigation into cancer in Norfolk. Annals of Internal Medicine. 2004;141(6):413–20. 15381514 10.7326/0003-4819-141-6-200409210-00006

[39] Carson AP, Reynolds K, Fonseca VA, Muntner P. Comparison of A1C and fasting glucose criteria to diagnose diabetes among US adults. Diabetes Care. 2010;33(1):95–7. 10.2337/dc09-1227 19808920 PMC2797994

[40] Connor JM, Millar SR, Buckley CM, Kearney PM, Perry IJ. The Prevalence and Determinants of Undiagnosed and Diagnosed Type 2 Diabetes in Middle-Aged Irish Adults. PloS one. 2013;8(11):e80504 10.1371/journal.pone.0080504 24282548 PMC3840064

[41] Balanda KP, Buckley CM, Barron SJ, Fahy LE, Madden JM, Harrington JM, et al Prevalence of Diabetes in the Republic of Ireland: Results from the National Health Survey (SLAN) 2007. PloS one. 2013;8(10):e78406 Epub 2013/10/23. 10.1371/journal.pone.0078406 24147134 PMC3797781

[42] Morris D, Khunti K, Achana F, Srinivasan B, Gray L, Davies M, et al Progression rates from HbA1c 6.0–6.4% and other prediabetes definitions to type 2 diabetes: a meta-analysis. Diabetologia. 2013;56(7):1489–93. 10.1007/s00125-013-2902-4 23584433

[43] Mainous AG, Tanner RJ, Baker R, Zayas CE, Harle CA. Prevalence of prediabetes in England from 2003 to 2011: population-based, cross-sectional study. BMJ open. 2014;4(6):e005002 10.1136/bmjopen-2014-005002 24913327 PMC4054625

[44] Yudkin JS, Montori VM. The epidemic of pre-diabetes: the medicine and the politics. Bmj. 2014;349:g4485 10.1136/bmj.g4485 25028385 PMC4707710

[45] Leahy S, Nolan A, O'Connell J, Kenny RA. Obesity in an ageing society: implications for health, physical function and health service utilisation. Dublin: TCD 2014.

[46] Hinchion R, Sheehan J, Perry I. Primary care research: patient registration. Ir Med J. 2002;95(8):249- 12405505

[47] Cronin S, Berger S, Ding J, Schymick JC, Washecka N, Hernandez DG, et al A genome-wide association study of sporadic ALS in a homogenous Irish population. Human molecular genetics. 2008;17(5):768–74. 18057069 10.1093/hmg/ddm361

[48] Wareham NJ, Griffin SJ. Should we screen for type 2 diabetes? Evaluation against National Screening Committee criteria. BMJ: British Medical Journal. 2001;322(7292):986 11312236 10.1136/bmj.322.7292.986 PMC1120142

[49] Khunti K, Davies M. Should we screen for type 2 diabetes: Yes. BMJ: British Medical Journal. 2012;345. 10.1136/bmj.e4514 22777029

[50] Organization WH. Screening for type 2 diabetes: report of a World Health Organization and International Diabetes Federation meeting: World Health Organization; 2003.

